# Analysis on the Economic Recovery in the Post-COVID-19 Era: Evidence From China

**DOI:** 10.3389/fpubh.2021.787190

**Published:** 2022-01-24

**Authors:** Dayang Jiang, Xinyu Wang, Rui Zhao

**Affiliations:** ^1^School of Economics, Tianjin University of Commerce, Tianjin, China; ^2^Faculty of Geosciences and Environmental Engineering, Southwest Jiaotong University, Chengdu, China

**Keywords:** COVID-19, economic development, resilience index, economic resilience, ARIMA model

## Abstract

As a major public health emergency, the COVID-19 pandemic has had a huge impact on economies all over the world. The experience of post-COVID-19 economic recovery is of great significance for achieving sustainable and high-quality economic development. Taking the economic development of China as an example, based on the theory of resilient economy and related measurement methods, this article selects five major indicators that are generally recognized as closely connected with economic resilience to construct a system of economic resilience indicators. In addition, the autoregressive integrated moving average (ARIMA) model is used to predict gross domestic product (GDP) under the scenario of no epidemic. The actual value of China's GDP is compared with the predicted value in the absence of the epidemic, verifying that strong economic resilience plays an important role in the country's economic response to major shocks. Based on the results, policy recommendations are made for countries to strengthen their economic resilience in the postepidemic era.

## Introduction

The outbreak of COVID-19 in early 2020 and its rapid spread across the globe have caused huge shocks to the economies around the world. The Chinese government takes timely and effective measures. By the end of the first quarter of 2020, the outbreak of China has been effectively controlled and the national economy has entered the postepidemic recovery period. According to the data released by various sources, major economies in the world except China showed negative growth in 2020. Among them, the economic growth rate of the United States was −3.5%, that of Japan was −4.8%, that of Germany was −5.0%, that of France was −9.0%, and that of Britain was −10.0%. What can be drawn from the unique economic recovery experience of China?

As early as the 1970s, inspired by the research of Holling (1) on the rapid self-healing ability of ecosystems after encountering natural or manmade interference, referred to as “resilience,” scholars further applied this concept into many other fields, namely, ecology, engineering, and economics. The application of this notion in economics was marked by the research of Martin (2) on the problem of measuring recovery and resistance index, which further provides strong support for the research on assessing the sensitivity of various regions to shocks and their response. Subsequently, Baltagi (3), Brada (4), and Oliva (5) discussed the regional economic resilience after major economic impact events such as external shocks (2008 financial crisis) and natural disasters (Japan earthquake), respectively. The frequent occurrence of external events makes the implications of research on economic resilience more relevant.

In response to the outbreak of COVID-19, the Chinese government took various policies and programs to maintain economic activities and even to achieve economic growth. The technologically driven pandemic-control programs such as the use of facial recognition technology and big data to track population flow and population spatial distribution provide opportunities for further application of these cutting-edge new technologies into industries. The international cooperation initiatives of the Chinese government such as the provision of biomedical products and medical supplies to developing countries increase the visibility of Chinese products in these countries, which further promotes China's exports. In terms of macroeconomic management, the Chinese government adopted a domestic demand-led growth approach to the unprecedented disruption that the COVID-19 pandemic has brought to world trade. Meanwhile, the Chinese government provided its manufacturing sector with strong fiscal incentives in promoting Chinese manufacturers to adapt their production capacity to the emerging demands for pandemic-control supplies. For instance, textile manufacturing plants were encouraged to devote their production capacity to masks and protective clothing production.

As the most populous country in the world, China has the largest labor force. The labor-intensive manufacturing sector of China has been serving as an essential driver of the country's growth for more than 4 decades. The various measures such as smart lockdown, public health interventions, and amendment of new laws regulations that the Chinese government took during the COVID-19 pandemic help control the spread of the COVID-19 and maintain the labor force of the country in good health condition. The stringency measures by the government (lockdown and COVID-19 testing requirement) might cause a sharp decline in economic activities in the short run. However, in the long run, stringency measures turn out to better help a country's economy recover from the shock of the pandemic. Meanwhile, the super large domestic market scale of China can be a great advantage for the country to promote the commercial use of the technological improvements (such as intelligent manufacturing) that have been achieved during the pandemic. Therefore, starting from the third quarter of 2021, the economy of China has experienced a rapid recovery.

According to the economic recovery of various countries in the post-COVID-19 period, this article aims to discuss the economic recovery of China by constructing a resilience index. The analysis results of the autoregressive integrated moving average model (ARIMA model) show that the economy of China has strong economic resilience. The article is organized as follows. The second section clarifies the definition of economic resilience and the mechanism of economic resilience under the impact of COVID-19 and makes the comparative analysis of various measurement methods of the economic resilience index. In the third section, the index measurement system for economic resilience is constructed, and the index is calculated and analyzed. The fourth section describes the setting of the ARIMA model, and this model uses an econometric model to make an empirical analysis of China's gross domestic product (GDP) data from 1990 to 2020. Finally, the fifth section makes predictions on the development trend of China's economy from the perspective of economic resilience.

## Economic Resilience Measurement

Economic resilience is the recovery ability of the economy aftershocks, which is developed based on ecological resilience. Edward (6) defines the resilience of the economic field as the ability of the regional economy to maintain or restore to a pre-existing state (usually assumed to be a balanced state) in the presence of certain types of exogenous (i.e., externally generated) shocks. Economic resilience is often dynamic. The speed at which an economy or system recovers from severe damage to an ideal state is an important indicator of the strength of economic resilience. Therefore, this article mainly analyzes the resilience of China's economy from the perspective of economic recovery after the COVID-19 outbreak.

**Figure 1:**
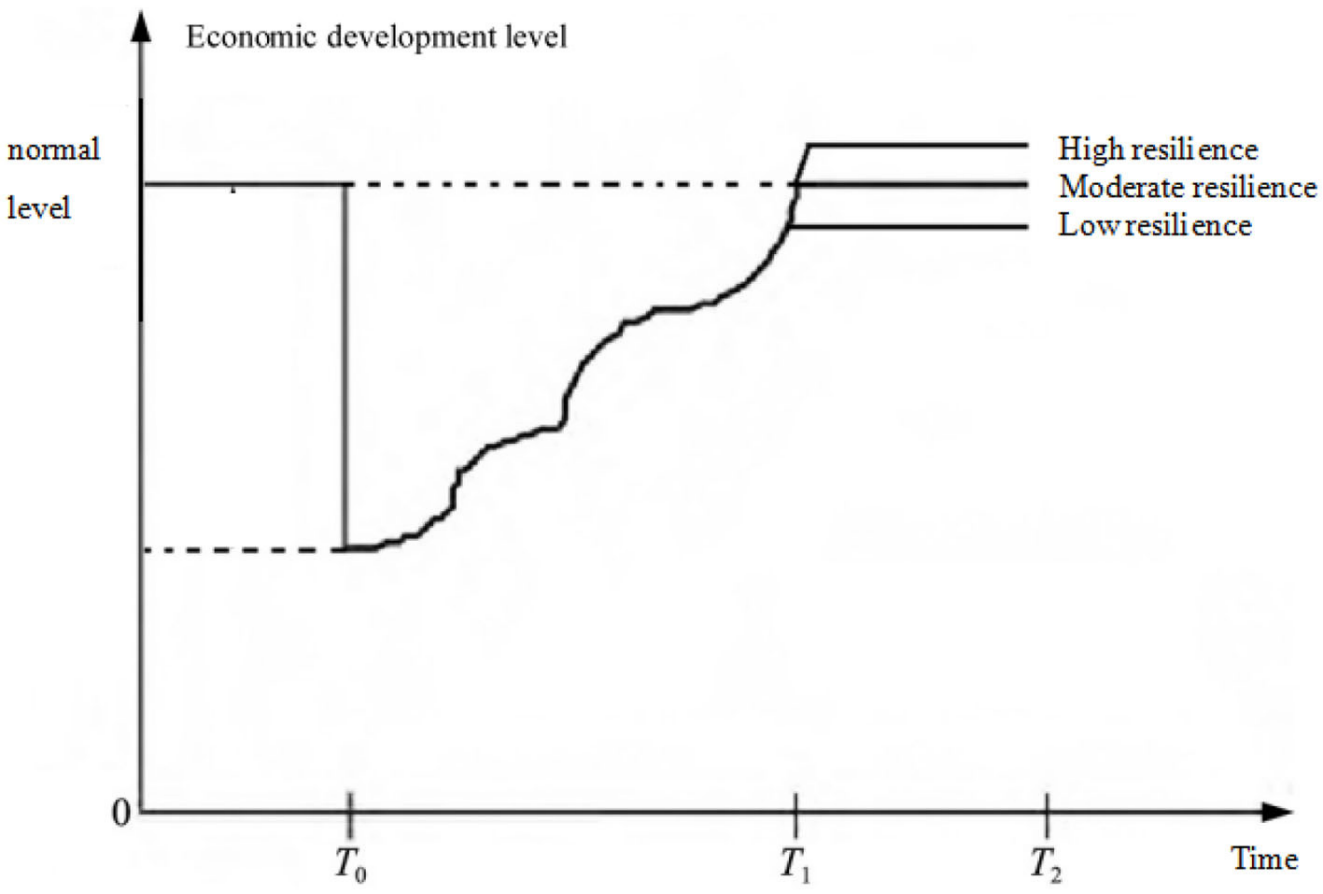
Economic development after the epidemic.

First, the path of economic development after the epidemic is sorted out in the light of Figure 1 to deepen understanding of economic resilience. The vertical axis represents the macroeconomic development level, which is a function changing with time. The lower the value is, the lower the economic development level is.

Assuming that the COVID-19 outbreak occurs at time *T*_0_, and the economic system operates at a normal level before *T*_0_. When the outbreak occurs, the level of economic development will be damaged to a certain extent. Then, with time, the economic system functions of the areas affected by the epidemic will continue to be repaired, and the economic recovery will be gradually realized at time *T*_1_ or *T*_2_. Moreover, from time *T*_0_ to time *T*_1_ or *T*_2_, the speed and degree of economic recovery are always in dynamic change. It is worth noting that Figure 1 only broadly divides the economic recovery from *T*_1_ to *T*_2_, focusing on showing the role of economic resilience in the process of economic recovery. In reality, there must be fluctuations between *T*_1_ and *T*_2_.

So far, scholars did not reach a consensus over the definition of economic resilience, especially for the resistance index and resilience index. However, following the original definition of ecological resilience (6), this article defines economic resistance as the ability of the economy to resist shocks. Resilience refers to the postshock resilience of an economy. As this article studies economic development after the epidemic, the measurement of resilience is more relevant from the perspective of economic recovery. Meanwhile, there are different measurement methods for economic resilience. For example, Briguglio et al. (7) adopted a multi-indicator comprehensive measure, which mainly measured economic resilience by constructing a measurement system covering macroeconomic stability, micromarket efficiency, economic governance, and social development. Martin (2) and Faggian et al. (8) used a single indicator (such as the unemployment rate) to measure the economic resilience after the impact of a crisis event.

This article fully absorbs the advantages of the two measurement methods. On the one hand, it draws on the comprehensive advantages of multi-index measurement, and selects five major indicators from the consumption, investment, import and export, government expenditure, and employment levels that are most closely related to the macroeconomy to establish an indicator evaluation system, and processes the data through entropy method.

First, the raw data are standardized,


$$\begin{array}{l} \text{Positive indicator }:\;{\text{X}}_{\text{ij}}^{*}=\frac{X_{ij}-\min\left(X_{j}\right)}{\max\left(X_{j}\right)-\min\left(X_{j}\right)}\\ \text{Negative indicator }:\;{\text{X}}_{\text{ij}}^{*}=\frac{\max\left(X_{j}\right)-X_{ij}}{\max\left(X_{j}\right)-\min\left(X_{j}\right)} \end{array}$$

With: $X_{ij}^{*}$ represents the standardized value of the *j*th index in the *i* quarter; *X*_*ij*_ represents the original index value of the *j*th index in the *i* quarter; max(*X*_*j*_) represents the maximum value of the *j*th indicator; min(*X*_*j*_) represents the minimum value of the *j*th indicator; i (*i* = 1, 2, 3, and 4) as the quarter; j (*j* = 1, 2, 3, 4, and 5) as a specific index.

Then, weights are given to each index. If the entropy value of the *j*th indicator is *e*_*j*_, then the information entropy redundancy of this indicator is *d*_*j*_ = 1 − *e*_*j*_, and the weight of each indicator is as follows: $a_{j}=\frac{1-e_{j}}{\sum_{j=1}^{5}d_{j}}$

Based on the above formula, the comprehensive evaluation value of each indicator is calculated as follows: $E_{i}=\overset{5}{\underset{j=1}{\sum }}a_{j}\times X_{ij}^{*}$.

On the other hand, the advantages of single index measure to fully consider the economic development in the crisis period and postcrisis period are learned, and the comprehensive indicators obtained by entropy method are analyzed and processed, to more accurately and specifically reflect the economic development in the post-COVID-19 era.

Therefore, this article will measure the economic resilience index according to the following formula:


$$\beta =(E_{i}\underset{t}{\;}-E_{i}\underset{t-\text{n}}{\;})/E_{i}\underset{t-n}{\;}$$

With:

*t* as for a quarter in 2020 and beyond; *t-n*, (*n* = 1,2) as for the corresponding quarter in 2019 and beyond; *E*_*i*_ represents the comprehensive evaluation value of the macroeconomy treated by the entropy method. β is the resilience index, the size of which represents the strength of economic resilience. The greater the restoring force index the larger the resilience index, the stronger economic resilience, and vice versa.

## Measuring Economic Resilience

According to the public data and statistical bulletins of the National Bureau of Statistics of China, the Ministry of Human Resources and Social Security, and the Ministry of Finance, the five major economic indicators from the first quarter of 2019 to the first quarter of 2021 were selected, namely, the number of new jobs in cities and towns, the national social total retail sales of consumer goods, import and export value, investment in fixed assets (excluding farmers), and the general public spending. An index evaluation system reflecting macroeconomic resilience is constructed, and the entropy method is used to comprehensively process the data. The weight assignment results and the influence direction of each index on macroeconomic are shown in Table 1.

**Table 1:** 2019 Q1–2020 Q1 economic data tables.

**Time**	**The number of employment of urban areas^**+**^(ten thousand people)**	**Total retail sales of social consumer goods in China^**+**^(One hundred million yuan)**	**Total import and export value^**+**^(thousand dollars)**	**Fixed-asset investment (excluding rural households)^**+**^(One hundred million yuan)**	**National general public budget expenditure^**+**^(One hundred million yuan)**
Q1 2019	324	97789.7	1027148357	101871	53656
Q2 2019	413	97420	1133999461	197229	54190
Q3 2019	360	101464.5	1190633071	162104	42832
Q4 2019	255	114974.8	1224345153	90274	39704
Q1 2020	229	78579.7	943006086	84145	45984
Q2 2020	335	93676.5	1086686340	197458	50192
Q3 2020	334	101067.8	2210049070	154927	41893
Q4 2020	288	118656.6	2436208317	90740	44826
Q1 2021	297	105220.8	1303602300	95994	57115
Weight	0.124	0.091	0.299	0.276	0.209

*Sign “+” represents positive influence; “-” indicates negative influence*.

In the postepidemic era, economic recovery is the primary task of the macroeconomy. Due to the significant seasonal differences in the data indicators, the economic resilience after the epidemic is measured quarterly. The calculation results are shown in Table 2.

**Table 2:** Measurement results of economic resilience from the first quarter of 2020 to the first quarter of 2021.

**Time**	**Comprehensive score**	**Resilience index (Economic resilience)**
Q1 2020	0.003	−0.986
Q2 2020	0.494	−0.198
Q3 2020	0.599	0.575
Q4 2020	0.655	1.704
Q1 2021	0.257	13.683

**Figure 2:**
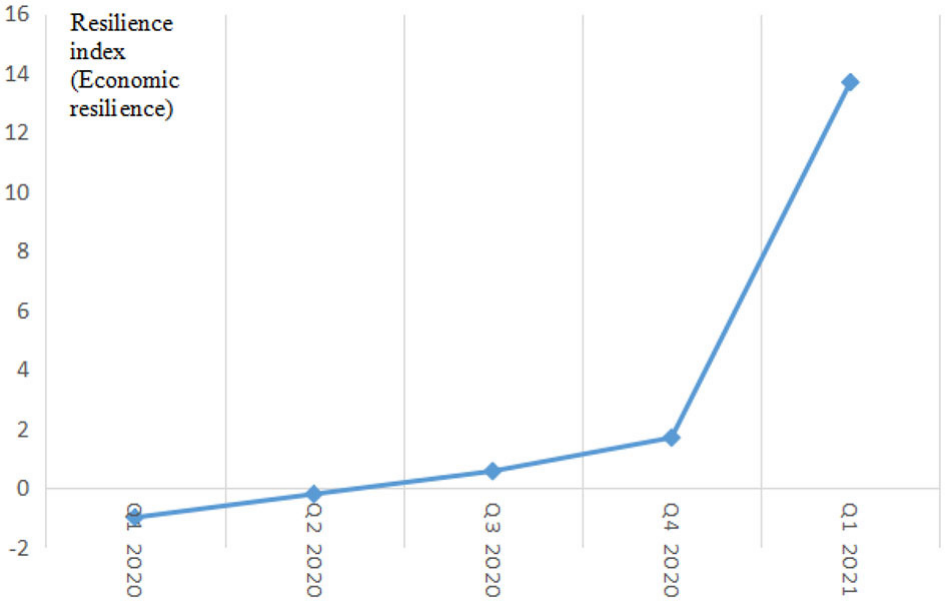
Line chart of China's macroeconomic resilience index from the first quarter of 2020 to the first quarter of 2021.

Figure 2 shows the line chart of China's macroeconomic Resilience Index. In addition, to ensure the effectiveness of the evaluation system constructed, we have conducted a robustness check. We use disposable national income per capita to replace the sales of consumer goods in China and add some additional relevant economic variables (such as the GDP index variable and the number of industrial enterprises). This robustness check shows that the trend of the resilience index has not changed, which fully shows that the results obtained by using the variables selected are reliable.

As a whole, the Chinese economy is greatly affected by the epidemic in the first half of 2020, and the resilience index is less than zero. However, the impact of the epidemic on the economy was short-lived. The resilience index began to reverse in the third quarter, showing a trend of substantial growth above zero. Economic resilience has effectively alleviated external shocks, and economic development has improved significantly.

Specific analysis shows that the first quarter of 2020 is the most severe period of the COVID-19 in China. A number of “rigid” epidemic prevention and control measures, such as city lockdown, regional isolation, and import and export restrictions, have damaged the overall environment for economic operation. In particular, the manufacturing and service industries have been severely affected by the epidemic, and the impetus for economic growth has been severely reduced. At this stage, the impact of the epidemic on economic development far exceeds the limit of economic resilience, and the economy as a whole is in the stage of “weak” recovery, and the resilience index is bound to be the lowest in the whole year. The resilience index in the second quarter was −0.198. Although it was still negative, it was significantly higher than the −0.986 resilience index in the first quarter. This is due to the effective control of the epidemic caused by several epidemic prevention and control measures taken in the early period. The improvement of the local epidemic has eased the external pressure on economic recovery and created a stable external environment for the resumption of work and production and the implementation of economic recovery policies. At the same time, due to the rapid rise in emergency demand for medical protection materials and living supplies caused by the epidemic, the production capacity and creativity of the secondary and tertiary industries have been fully stimulated, and many new industries and new formats have been born and promoted, which has injected new vitality into the economic recovery after the epidemic. In the second half of 2020, COVID-19 has basically calmed down in China, and economic and industrial policies that have hedged the impact of the epidemic have been implemented. The Chinese economy has entered a new stage of restoration, the resilience index has been increasing, and the resilience of the national economy has become more evident. In addition, the complex industrial chain formed with the epidemic as the core has been continuously extended and optimized, which has opened up a new path for economic recovery. Since the third quarter, the economy of China has entered a period of rapid recovery, and the economic development trend has been improving.

## ARIMA Model

### Specification of the Model

Autoregressive integrated moving average (ARIMA) was first proposed by Box and Jenkins (1970). The basic idea of the model is to take the sequence formed by the change of the predictor variable over time as a random sequence, and then use a specific mathematical model to describe the random sequence based on the autocorrelation of the time sequence. In this article, the sequence of China's GDP data from 1990 to 2020 is taken as a random sequence, and the predicted GDP value is generated by the ARIMA model, and the effectiveness of the model is judged by the relative error between the predicted GDP value and the real GDP value, and the relative error of 2020 is compared with other years to explain the macroeconomic development of China under the epidemic situation. ARIMA (p, d, q) is a combination of autoregressive model, moving average model, and difference method, that is, the ARMA model after the difference of nonstationary series. This article constructs the ARIMA model as follows:


$$\begin{array}{r} y_{t}=\varphi_{1}y_{t-1}+\varphi_{2}y_{t-2}+\ldots +\varphi_{p}y_{t-p}+\varepsilon t-\theta_{1}\varepsilon_{t-1}\\ -\theta_{2}\varepsilon_{t-2}-\ldots -\theta_{q}^{\;}\varepsilon_{t-q} \end{array}$$

With:

*p* is the order of the autoregressive model;

*q* is the order of the moving average model;

ϕ_*i*_(*i* = 1, 2, …, *p*) θ_*j*_ (*j* = 1, 2, …, *q*) is the undetermined coefficient of the model; and

ε_*t*_ is the residual; *y*_*t*_ is the predicted value.

### GDP Prediction Using ARIMA

**Figure 3:**
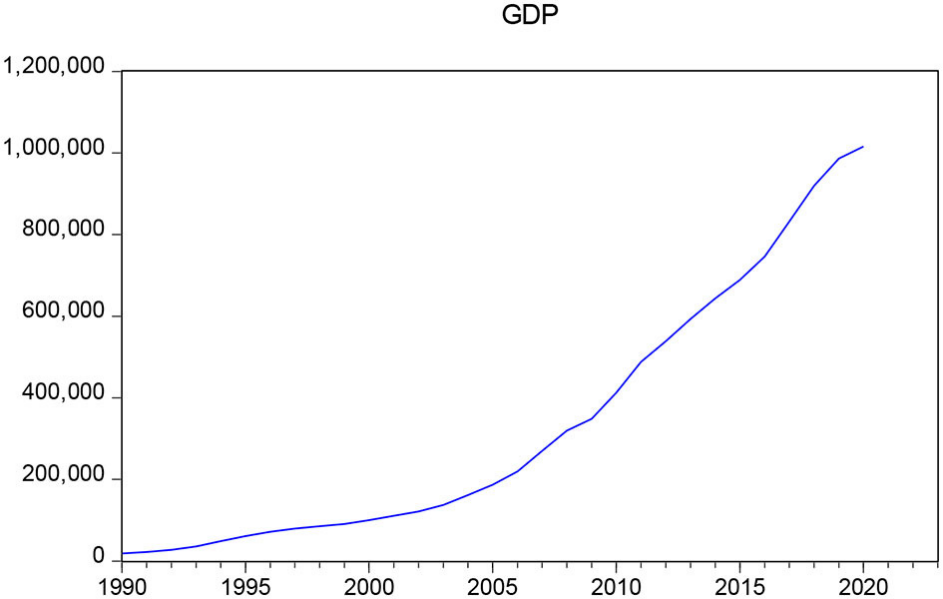
Time series chart of GDP data from 1990 to 2020.

The data selected in this article are Chinese GDP data from 1990 to 2020. To ensure the accuracy and authenticity of the data, all the selected data are from the National Bureau of Statistics of China. As can be seen from Figure 3, the time-series data of GDP are nonstationary, which needs further processing. Therefore, the data are processed logarithmically, but it is found that the data are still obviously nonstationary, and then the data are subjected to a second difference, and the obtained data have no obvious trend. The augmented Dickey–Fuller (ADF) test is performed on the processed data, as shown in Table 3. The t-statistic is −4.946446, which is far less than the critical value of 5% and 1% significance level, and the *p* value is 0, indicating that the data have stabilized.

**Table 3:** ADF unit root test in second-order difference.

**Variables**	**t-statistics**	Prob.^*^	**Critical value at 1%**	**Critical value at 5%**	**Critical value at 10%**
GDP	−4.946446	0.0023	−4.323979	−3.580623	−3.225334

* *Refers to Mackinnon (1996) one-sided p-values*.

**Figure 4:**
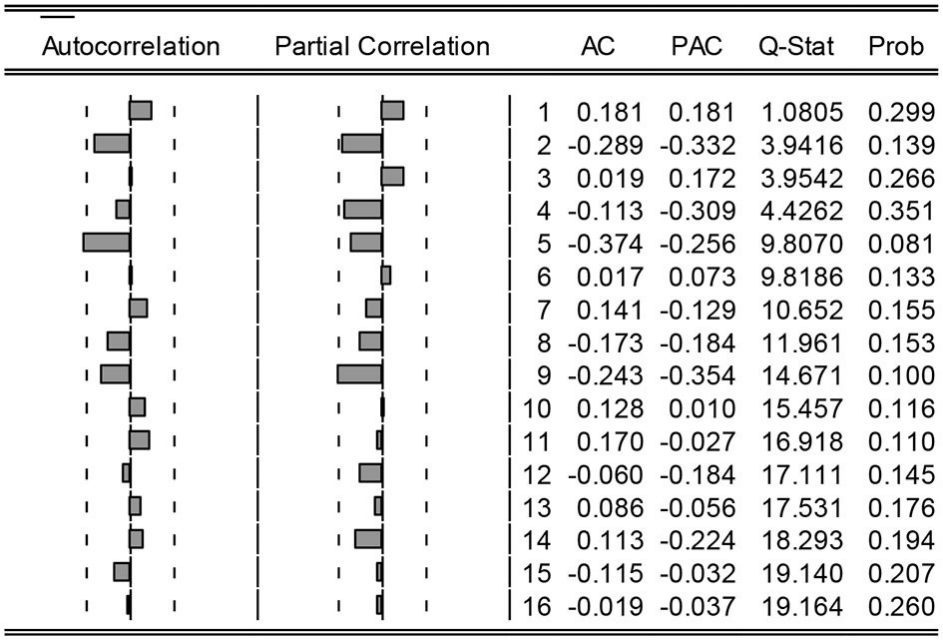
Autocorrelation and partial autocorrelation graph.

The autocorrelation and partial autocorrelation diagrams of the processed GDP data are shown in Figure 4. The partial autocorrelation coefficients of the data are obviously truncated, so try ARIMA (1, 1, 1), ARIMA (0, 1, 1), and ARIMA (0, 1, 2) and other models. By comparing each model, it is found that only the parameter estimates of the explanatory variables of the ARIMA (0, 1, 1) model are significant at the 5% significance level, and the Akaike information criterion (AIC) and Bayesian Information Criterion (SBC) of the model are smaller than other models. The fitting results of the model are shown in Table 4.

**Table 4:** ARIMA (0, 1, 1) model fitting results.

**Variable**	**Coefficient**	**Std. error**	**t-statistic**	**Prob**.
MA (1)	0.660252	0.132917	4.967392	0.0000
R-squared	0.174053	Mean dependent var		−0.004280
Adjusted R-squared	0.174053	S.D. dependent var		0.044004
S.E. of regression	0.039992	Akaike info criterion		−3.566410
Sum squared resid	0.044782	Schwarz criterion		−3.519261
Log likelihood	52.71294	Hannan-Quinn criter.		−3.551643
Durbin-Watson stat	2.324238			
Inverted MA Roots	−0.66			

**Figure 5:**
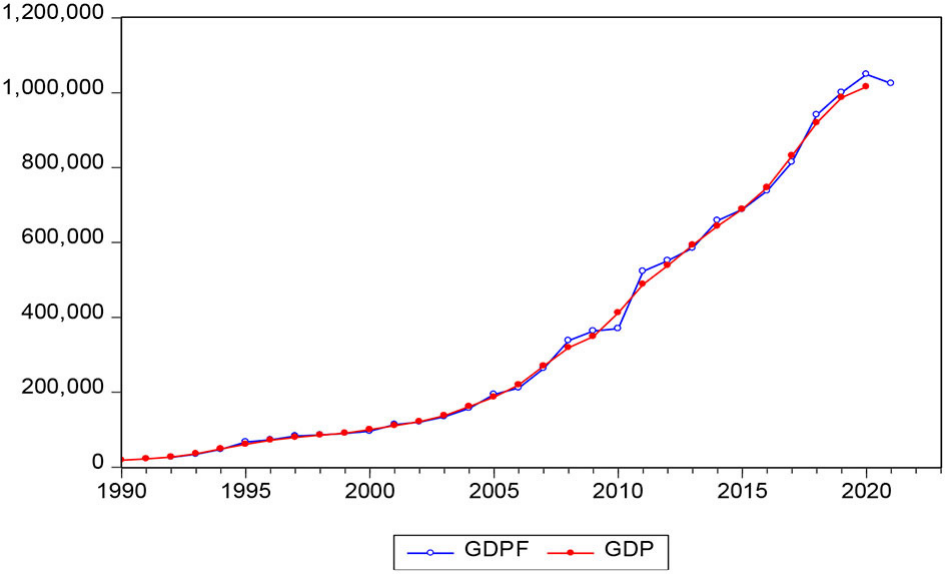
Fitting graph of Chinese GDP forecast value and actual value from 1990 to 2020.

It can be seen from Figure 5 that the predicted value obtained in the ARIMA (0, 1, 1) model is very close to the real value, and the fitting effect is better. To further test the model fitting quality, this article calculates the relative error between the real GDP value and the predicted GDP value from 1992 to 2020. It is found that the relative error of only 3 years is >5%, and the relative error of the other years is within 5%, with an average of 3.13%. The small error fully demonstrates the high accuracy of the model prediction. It is worth noting that the impact of COVID-19 on the economy is more severe than any major natural disaster or financial crisis in the past, but the relative error between the actual value of Chinese GDP in 2020 and the predicted value under the condition of no epidemic intervention is only 3.14%, and the level is equal to the average level, which fully demonstrates the strong economic resilience of China.

## Conclusions

It is a consensus that COVID-19 has an impact on the economy of all countries. This article mainly analyzes Chinese economic recovery after the outbreak of the epidemic. First of all, by measuring the economic resilience from the first quarter of 2020 to the first quarter of 2021, we find that the economic resilience index only shows a negative value in the first two quarters of 2020, while the resilience index shows a relatively strong growth momentum in the third and fourth quarters, which fully highlights the strong economic resilience of China. Therefore, the Chinese economy can achieve recovery and growth in a relatively short period. Second, the ARIMA model is used to forecast GDP, and we find that the actual value of Chinese GDP in 2020 is not much different from the predicted value (in the absence of the epidemic), which further confirms the above judgment. To sum up, we can conclude that COVID-19 did have an impact on the economy of China, but strong economic resilience has prompted the rapid recovery of the Chinese economy after the epidemic, with a higher degree of recovery.

Finally, learning from the postepidemic recovery of China, there is no doubt that strengthening economic resilience is an effective way to cope with and mitigate such external shocks, and favorable support for the economic recovery of all countries. On one hand, COVID-19 and natural disasters are external shocks, which affect the operating environment of the economy and cause damage to economic development. The establishment of a more complete risk emergency mechanism and social governance system can ensure the stability of the economic operating environment and promote economic resilience, and thus create conditions for economic recovery. On the other hand, scientific and sound macroeconomic policies are an important aspect of enhancing economic resilience. The government should enhance the ability of dynamic adjustment, fully mobilize the enthusiasm and vitality of supply and demand sides through policy guidance, and improve the ability of each economy to cope with risks.

## Data Availability Statement

The original contributions presented in the study are included in the article/supplementary material, further inquiries can be directed to the corresponding author.

## Author Contributions

DJ: conceptualization, methodology, formal analysis, writing—original draft preparation, and funding acquisition. XW: formal analysis and writing—original draft preparation. RZ: conceptualization, methodology, and project management. All authors have read and agreed to the published version of the manuscript.

## Funding

This authors acknowledge financial support from Social Science Fund of Municipal Education Commission of Tianjin, China (Grant#: 2019JWZD56).

## Conflict of Interest

The authors declare that the research was conducted in the absence of any commercial or financial relationships that could be construed as a potential conflict of interest.

## Publisher's Note

All claims expressed in this article are solely those of the authors and do not necessarily represent those of their affiliated organizations, or those of the publisher, the editors and the reviewers. Any product that may be evaluated in this article, or claim that may be made by its manufacturer, is not guaranteed or endorsed by the publisher.
